# Unveiling the Mechanistic Singularities of Caspases: A Computational Analysis of the Reaction Mechanism in Human Caspase-1

**DOI:** 10.1021/acscatal.3c00037

**Published:** 2023-03-15

**Authors:** Carlos A. Ramos-Guzmán, J. Javier Ruiz-Pernía, Kirill Zinovjev, Iñaki Tuñón

**Affiliations:** † Departamento de Química Física, Universitat de Valencia, 46100 Burjassot, Spain; ‡ Instituto de Materiales Avanzados, Universitat Jaume I, 12071 Castelló, Spain

**Keywords:** caspase-1, cysteine proteases, CD clan, reaction mechanism, minimum free energy path, QM/MM, string method

## Abstract

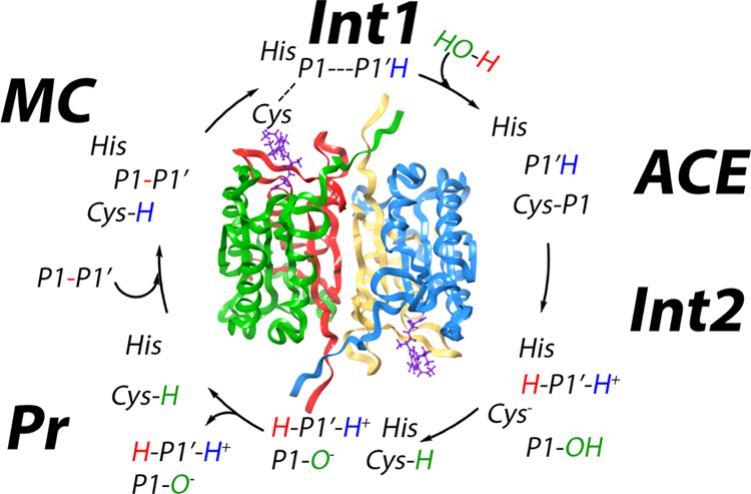

Caspases are cysteine proteases in charge of breaking a peptide bond next to an aspartate residue. Caspases constitute an important family of enzymes involved in cell death and inflammatory processes. A plethora of diseases, including neurological and metabolic diseases and cancer, are associated with the poor regulation of caspase-mediated cell death and inflammation. Human caspase-1 in particular carries out the transformation of the pro-inflammatory cytokine pro-interleukin-1β into its active form, a key process in the inflammatory response and then in many diseases, such as Alzheimer’s disease. Despite its importance, the reaction mechanism of caspases has remained elusive. The standard mechanistic proposal valid for other cysteine proteases and that involves the formation of an ion pair in the catalytic dyad is not supported by experimental evidence. Using a combination of classical and hybrid DFT/MM simulations, we propose a reaction mechanism for the human caspase-1 that explains experimental observations, including mutagenesis, kinetic, and structural data. In our mechanistic proposal, the catalytic cysteine, Cys285, is activated after a proton transfer to the amide group of the scissile peptide bond, a process facilitated by hydrogen-bond interactions with Ser339 and His237. The catalytic histidine does not directly participate in any proton transfer during the reaction. After formation of the acylenzyme intermediate, the deacylation step takes place through the activation of a water molecule by the terminal amino group of the peptide fragment formed during the acylation step. The overall activation free energy obtained from our DFT/MM simulations is in excellent agreement with the value derived from the experimental rate constant, 18.7 vs 17.9 kcal·mol^–1^, respectively. Simulations of the H237A mutant support our conclusions and agree with the reported reduced activity observed for this caspase-1 variant. We propose that this mechanism can explain the reactivity of all cysteine proteases belonging to the CD clan and that differences with respect to other clans could be related to the larger preference showed by enzymes of the CD clan for charged residues at position P1. This mechanism would avoid the free energy penalty associated with the formation of an ion pair. Finally, our structural description of the reaction process can be useful to assist in the design of inhibitors of caspase-1, a target in the treatment of several human diseases.

## 1 Introduction

Cysteine-dependent aspartate-specific proteases, or caspases, are very important pharmacological targets since they are involved in processes such as inflammatory cytokines maturation and cellular apoptosis.^1^ Some relevant human diseases such as rheumatoid arthritis,^2^ acute brain injury,^3^ epilepsy,^4^ and Alzheimer’s disease^5^ are related to caspases inflammation dysregulation.^6^ On the other hand, deregulated apoptosis can lead to numerous types of cancer.^7^ Among the human caspase family, at least 12 members have been identified,^8,9^ being caspases 1, 4, and 5 involved in the inflammatory response. Regarding the apoptotic group, it is divided into initiator and executioner/effectors enzymes. Caspases 8, 9, and 10 belong to the former, while caspases 3, 6, and 7 belong to the latter.^9^ In addition to their role as inflammatory and apoptotic enzymes, some caspases have cell-cycle-related functions, such as caspase-2, or cell differentiation role, as in the case of caspase-14. So far, the function of caspase-12 remains undefined.^9,10^

**Figure 1:**
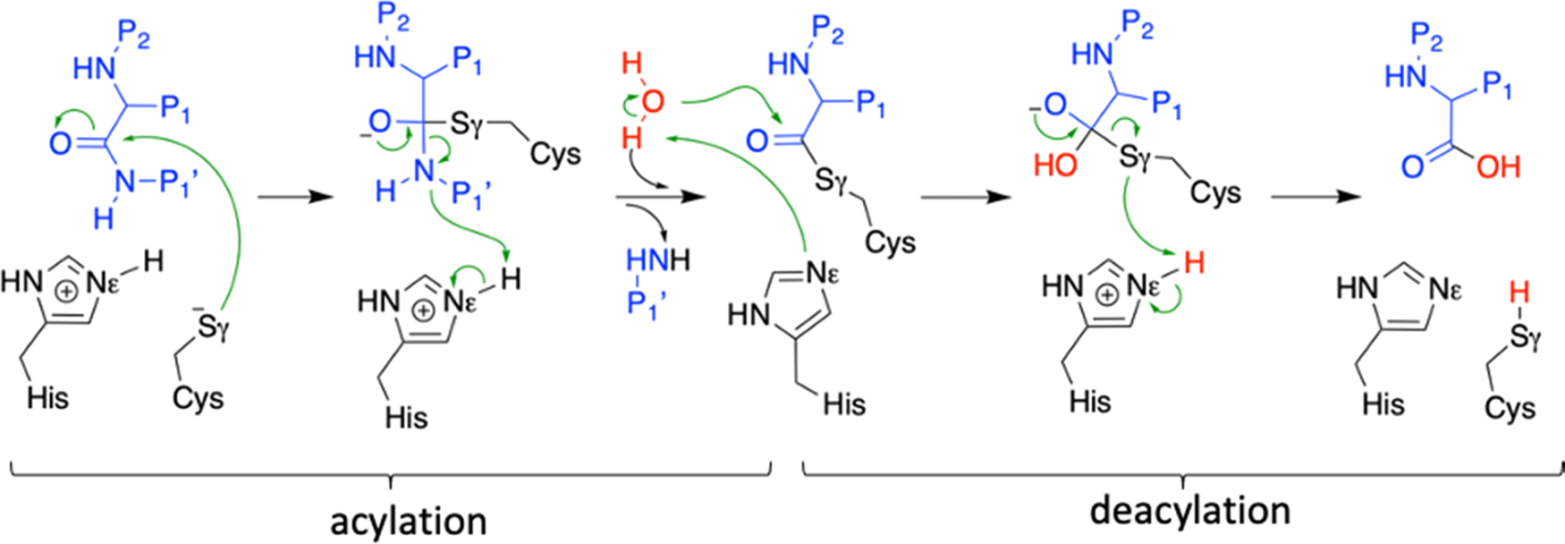
Putative reaction mechanism for caspase enzymes. In human caspase-1, the catalytic dyad is formed by His237 and Cys285.

Despite their different roles, all of the caspases share a similar heterodimeric structure, amino acid sequence, and the same substrate specificity for an aspartate residue (Asp) in the P1 position of the peptidic substrate.^10,11^ P*i* and P*i*′, respectively, denote the *i*-position before and after the scissile peptide bond, the bond formed between the P1 aspartate and the amino acid in the P1′ position. The standard reaction mechanism proposed for these enzymes, as for other cysteine proteases, is divided into two separated steps, acylation and deacylation (see Figure 1).^12^ During the acylation step, the Sγ atom of the catalytic cysteine (Cys285 in caspase-1 numbering) would perform the nucleophilic attack on the carbonyl carbon of the P1 residue of the substrate (C(P1)), forming a tetrahedral intermediate. This intermediate would then abstract a proton from the catalytic histidine (His237 in caspase-1) to be transformed into the acylenzyme complex after releasing the amino leaving group (P1′-NH_2_ in Figure 1). During the second step, deacylation, the acylenzyme complex would be hydrolyzed by a water molecule activated by the catalytic histidine, forming a new tetrahedral intermediate from which the enzyme is regenerated and the product released.

**Figure 2:**
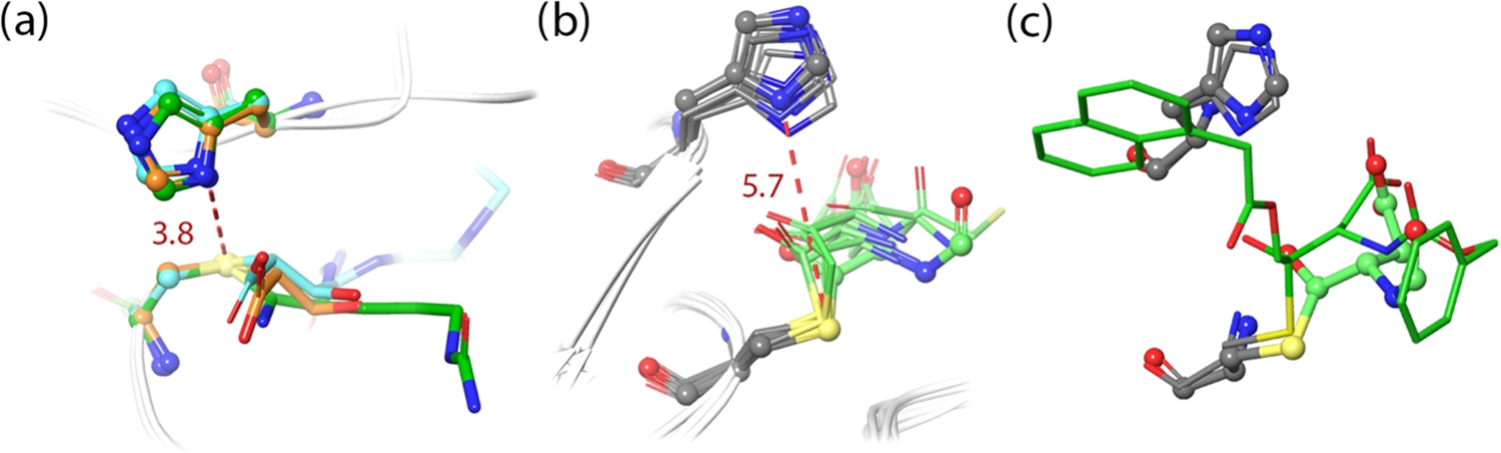
X-ray structures for the acyl–enzyme complex of some cysteine proteases. (a) Papain in cyan, actinidin in orange, and cathepsin B in green (PDB codes: 1PPP, 1AEC, and 6AY2, respectively) protein atoms in ball representation and substrate in licorice. (b) Caspase-1, -2, -3, -6, -7, -8, and -9 (PDB codes: 6F6R, 1PYO, 1NME, 3OD5, 3IBC, 1QDU, and 1JXQ, respectively). Average donor–acceptor distances are given in angstrom. (c) Apo caspase-1 (PDB code 6F6R, ball and rod representation) and the covalent complex formed with **4i** (in licorice).

The knowledge of mechanistic details is important for the design of enzymatic inhibitors that could be potentially used as drugs to regulate caspase activities. For example, transition and intermediate states along the reaction path can be used as templates for the development of new enzymatic inhibitors.^13^ However, the standard mechanistic proposal for cysteine proteases presents some difficulties in the case of caspases. The overall mechanism presented in Figure 1 surmises a thiolate–imidazolium ion-pair configuration for the catalytic dyad in the noncovalent enzyme–substrate or Michaelis complex. However, this ion pair configuration of the catalytic dyad has been discarded in the study of the complexes formed between inhibitors with caspase-3,^14^ caspase-7,^15^ and caspase-1.^16^ Simulations of these complexes in the ion pair configuration of the catalytic dyad showed significant conformational rearrangements and a trend of the inhibitor to move away from the active site. Instead, the neutral configuration for the catalytic dyad is more likely to exist than the charged one at the resting state.^16^ Thus, the thiol group in the catalytic cysteine must be deprotonated/activated once the Michaelis complex is formed, not before. In other cysteine proteases, such as cathepsin B, actinidin, or papain, this activation is produced by means of a direct proton transfer from the cysteine to the histidine in the catalytic dyad, as these two residues are close enough accordingly to their crystallographic structures (see Figure 2a).^17−19^ However, for the case of caspases, the crystallographic distance between the proton donor and acceptor atoms is larger than 5 Å (Figure 2b).^20−26^ Moreover, the substrate is placed in between the catalytic dyad, as observed in the crystallographic structure of caspase-1 with compound **4i**.^27^ The same is observed for other not P1-Asp-specific cysteine protease members of the CD clan, such as leguamains,^28^ gingipains,^29^ and clostripains.^30^ These observations suggest that the direct proton transfer from cysteine to histidine in the catalytic dyad is not feasible for caspases and, therefore, a different activation path has to be envisaged, as proposed for other cysteine proteases of the CD clan.^31,32^

We focus our mechanistic research on the human enzyme caspase-1, an enzyme in charge of the transformation of the pro-inflammatory cytokine pro-interleukin-1β (pro-IL-1β) into its IL-1β active form, which plays a key role in the inflammatory response to infection, injury, or disease and can lead to cell death in mammals.^33,34^ The global *k*_cat_ values for the reaction of the caspase-1 enzyme with substrates Ac-YVAD-pNA and Ac-YVAD-mNA at 303 K are 0.78 and 0.88 s^–1^, respectively,^35^ which according to transition state theory corresponds to an activation free energy barrier of about 17.9 kcal·mol^–1^. The activity of caspase-1 processing its natural substrate (pro-IL-1β) has been studied for the wild-type enzyme and different mutants. It was observed, not surprisingly, that the C285S mutant is completely inactive.^36^ Nevertheless, cells containing the H237A, H237K, and H237Q mutants have been shown to still produce IL-1β, around 9–15% of the amount produced by cells containing the wild-type variant after 24 h, which indicates that these mutants retain some enzymatic activity.^36^ This observation questions the key role assigned to the histidine residue of the catalytic dyad, transferring a proton to the amino leaving group, as assumed in the standard mechanism (see Figure 1). An alternative role for His237 was proposed by Brady and collaborators,^37^ after studying the inhibition of caspase-1 by activated aspartic ketones via a thiohemiketal complex. Based on the distances observed between the Nδ atom of the imidazolium ring and the carbonyl oxygen atom of the P1 group, these authors hypothesize that the Nδ atom polarizes this group during the acylation step, facilitating the nucleophilic attack to the electrophilic carbon atom. Nevertheless, in some crystallographic structures of the inhibited enzyme, the observed distance between the His237-Nδ atom and this carbonyl oxygen atom of the substrate is larger (see Table S1) and the oxygen atom becomes closer to the amino groups forming the oxyanion hole (Cys285 and Gly238). In this way, the proposal that His237 polarizes the carbonyl group of the scissile peptide bond remains inconclusive.

To our knowledge, the reaction mechanism of caspase-1 has not been computationally analyzed to date. The acylation reaction in caspase-7 was studied by Miscione et al.^15^ using a cluster model of the active site and an inhibitor. In their mechanistic proposal, the catalytic cysteine is activated after a proton transfer to the backbone amino group of the P1 aspartate, whose proton was previously abstracted by the carboxylate group of the side chain. Then, after a series of proton transfers, a water molecule protonates the amino leaving group of the P1′ residue, assisted by the catalytic histidine, and the acylenzyme complex is formed. A 25.2 kcal·mol^–1^ reaction barrier was reported for this step, significantly larger than the value derived from the experimental overall rate constant for a caspase-7 (17.7 kcal·mol^–1^).^38^ The observed reaction mechanism is probably a consequence of the reduced reaction model. The inhibitor contains an aldehyde group in the P2 position, resulting in a small and too flexible C-terminal fragment. Also, the presence of several water molecules is probably due to the absence of the N-terminal fragment in the model, as the active site should remain more inaccessible to water molecules when the P1′ fragment is present. The deacylation step was studied by Sulpizi et al.^39^ in caspase-3 using a QM/MM approach. In their mechanistic proposal, the acylenzyme complex is hydrolyzed by a water molecule activated by the catalytic histidine (as shown in Figure 1). The activated water molecule then attacks the carbonyl carbon and the catalytic histidine protonates the oxygen of the carbonyl group of the P1 residue, forming a gem-diolate intermediate before the release of the products. However, this mechanistic hypothesis for the deacylation step does not explain the reduced enzymatic activity still observed for mutants where the catalytic histidine is substituted by a nonbasic residue, such as alanine.^36^

So far, a complete description of the whole reaction mechanism for caspases, compatible with experimental evidence is not available. In this work, we afford the study of the mechanism of caspase-1 using a hybrid density functional theory/molecular mechanics (DFT/MM) potential and the adaptive string method^40^ for the exploration of the multidimensional free energy landscape. From the analysis of our simulations, we propose a mechanism for the proteolytic activity of caspase-1 compatible with structural, kinetic, and mutagenesis data and that can be extrapolated to other members of the caspase family. Our proposal offers an alternative to the activation of the catalytic cysteine without the participation of the catalytic histidine. We also propose a role for this histidine, which we corroborate by performing simulations on the H237A mutant. In addition, the calculated activation free energies for the wild-type and mutant variants of caspase-1 are in very good agreement with the experiments. To our knowledge, this is the first time that a complete mechanistic picture explaining the singularities of caspases is provided. This study also identifies several intermediate states that could be useful as templates for the design of caspase-1 inhibitors, a subject of great pharmaceutical interest due to the implications of this enzyme in inflammatory processes.

## 2 Methods

### 2.1 Classical Molecular Dynamics Simulations

**Figure 3:**
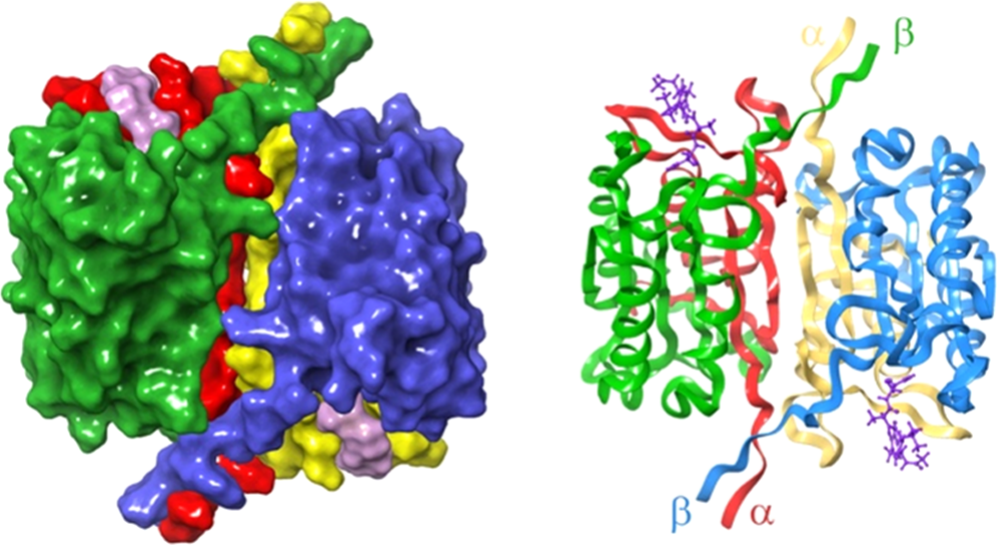
Representation of the 1ICE crystallographic structure for caspase-1. Left: surface representation of the homodimer formed by two heterodimers. One monomer shows subunits α and β colored in red and green, respectively; the other monomer presents subunits α and β in yellow and blue, respectively. The co-crystallized inhibitor in the active site is colored purple. Right: ribbon representation of the αββ′α′ dimeric structure using the same colors code.

Caspase-1 is a dimeric enzyme with two subunits, α and β.^36^ The residues forming the active site come from both α and β subunits. In the active form, caspase-1 forms a dimer of dimers in an αββ′α′ symmetry, having in this way two active sites per molecule (see Figure 3).^1^ Experimental data suggest that the occupation of one active site promotes the activity in the second one.^41^

To build a model of the Michaelis complex formed by the caspase-1 enzyme and a peptidic substrate, two crystallographic structures with PDB codes 6F6R^21^ and 1ICE^36^ were employed. The protein structure was selected from 6F6R because this structure contains a larger number of resolved residues in the β subunit, while the coordinates of the co-crystallized peptide-like inhibitor, with sequence Ac-Tyr-Val-Ala, were taken from the 1ICE. The co-crystallized inhibitor was elongated using the Maestro tool^42^ building the sequence Ac-Tyr-Val-Ala-Asp-Ala-Pro-Val-Arg-NMe. The substrate was placed in both dimers, as some studies suggest that the activity of the enzyme is increased when both active sites are occupied^41^ and also because the inhibitor is present in both active sites of the X-ray structures of the heterodimers.^20,36,43,44^ Hydrogen atoms were added using the protein preparation wizard tool of Maestro. To determine the more likely protonation state of the titratable residues at pH 7.4, PROPKA3.0^45^ was used. According to this, the catalytic dyad was initially modeled as neutral, with His237 protonated at Nε, although the ion pair (IP) was also considered, see below. In a previous study about the noncovalent complex formed between caspase-1 and several inhibitors, we showed that only the simulation of a neutral His237 protonated at Nε explains the experimental trends and the observed short distance between this atom and the carbonyl group of Pro177 in X-ray structures.^16^ The force field used to describe the amino acid residues was the ff14SB.^46^ The protein was solvated in a cubic box of flexible TIP3P^47^ water molecules assuring that there were no protein/substrate atoms closer to 12 Å to the limits of the box. Two Na^+^ ions were also added to neutralize the charge of the system. The system was built with tleap tool from the Ambertools package.^48^

The initial geometry of the system was minimized using 500 steps of the steepest descent method followed by the conjugate gradient algorithm until the root-mean-square of the gradient was below 10^–3^ kcal·mol^–1^·Å^–1^. The system was then heated from 0 to 300 K during 120 ps, using a linear heating ramp while the positions of the heavy atoms of the protein backbone were restrained using a harmonic force constant of 20 kcal·mol^–1^·Å^–2^. The system run another 20 ps once the temperature reached 300 K. Then, 7.5 ns of NPT (300 K and 1 bar) simulation were run to equilibrate the system. During this process, the harmonic force constant was decreased from 15 to 3 kcal·mol^–1^·Å^–2^ by 3 units every 1.25 ns, followed by a restraint-free simulation of 1.25 ns. From the final configuration, we ran three replicas of 1 μs of NVT simulation (starting from different initial velocities) at 300 K. The bonds involving hydrogen atoms were constrained using SHAKE,^49^ which allowed us to use a time step of 2 fs. The long-range electrostatic interactions were treated by the particle mesh Ewald method,^50,51^ and a cutoff of 10 Å was used for the short-range interactions. Temperature and pressure were controlled using the Langevin thermostat and the Berendsen barostat, respectively. All classical molecular dynamic simulations were made using the AMBER19 GPU version of pmemd^52,53^ employing periodic boundary conditions. The same procedure was employed to build a model of the Michaelis complex for the H237A mutant. In that case, residue H237 was transformed into alanine deleting the imidazolium ring.

### 2.2 QM/MM Calculations

**Figure 4:**
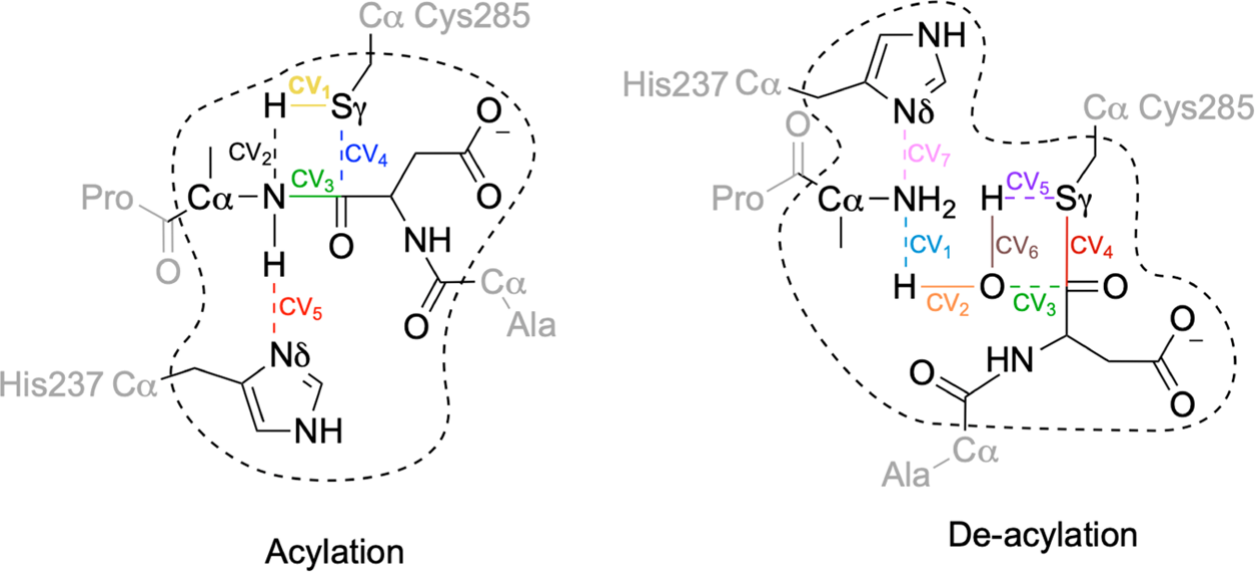
QM/MM partitioning scheme used to evaluate the acylation and deacylation reaction mechanisms. Atoms inside the dashed regions were treated at the QM level while the rest was evaluated at the MM level. The set of CVs describing the relevant changes during the chemical steps are also shown.

To determine the minimum free energy path (MFEP) for the acylation and deacylation steps in caspase-1 (wild-type and mutant variants), a quantum mechanics/molecular mechanics (QM/MM) treatment was employed. Here, the QM region contains the side chains of the catalytic dyad, Cys285 and His237 (Ala237 for the mutant), while for the substrate Asp-P1 and Ala-P1′, the residues forming the peptide bond to be broken and the contiguous peptide bonds up to the next Cα atoms were included in the QM subsystem. For the deacylation step, a water molecule was also included. The QM regions are shown in Figure 4 (see Figure S1 for the H237A mutant). The B3LYP^54,55^ functional with the 6-31G* basis set and D3 dispersion corrections^56^ was used to describe the QM subsystem. This combination was selected based on our previous experience with cysteine proteases,^57−61^ where we found that this functional provides free energy profiles in good agreement with experimental data. The simulations were run in a modified version of Amber18^48,62^ with Gaussian16.^63,64^ All simulations were run at 300 K with a cutoff radius of 15 Å for QM/MM electrostatic interactions.

The adaptive string method (ASM)^40^ was used to obtain the minimum free energy path (MFEP) on the multidimensional free energy landscape. This methodology was selected because both steps of the enzymatic reaction (acylation and deacylation) involve many degrees of freedom. In ASM, simulations are run over a series of replicas of the system (the string nodes) centered at different positions in the space of collective variables (CVs). The string nodes evolve toward lower free energy regions, while being evenly distributed along the string, which ensures its convergence to the MFEP. In each 50 simulation steps, a Hamiltonian replica exchange attempt between neighboring string nodes was made. Once the string has converged, a single path-CV, which is a function that increases monotonically when the system moves along the path, is used as a reaction coordinate for subsequent free energy calculations. A total of four string calculations were carried out in this work: acylation and deacylation steps for both the wild-type and mutant systems. The set of CVs used in this study to describe both chemical steps is described in Figure 4 (see Figure S1 for the study with the H237A mutant) and includes the distances of all of the bonds to be formed or broken during the process. Each string was composed of 96 nodes. The string was considered as converged when the RMSD of the string was less than 0.1 amu^1/2^·Å for more than 2 ps. 10 ps umbrella sampling^65^ windows were run for each string node to collect sampling along the path-CV. The values of the window positions and the force constants were determined on-the-fly during the string optimization to obtain a homogeneous probability density distribution along the reaction coordinate during umbrella sampling. Free energy profiles along the path-CV were obtained using WHAM.^66^ Hydrogen atoms being transferred during the reaction were given a mass of 2 amu and the time step used in all QM/MM simulations was 1 fs. Error intervals were determined as the standard deviation obtained from the bootstrap method.^67^ Note that the use of higher-level QM methods can reduce systematic error due to cheaper QM descriptions, but at the cost of reducing the sampling time, which in turn can result in an increase of statistical errors.

## 3 Results

### 3.1 Enzyme–Substrate Complex

The root-mean-square deviation (RMSD) of protein and substrate backbone atoms for the three 1 μs replicas of the wild-type enzyme and H237A mutant systems are shown in Figure S2. For all replicas, the RMSDs remained in the region of relatively small values, showing that there were no large conformational changes with respect to the X-ray structure during the simulations.

**Figure 5:**
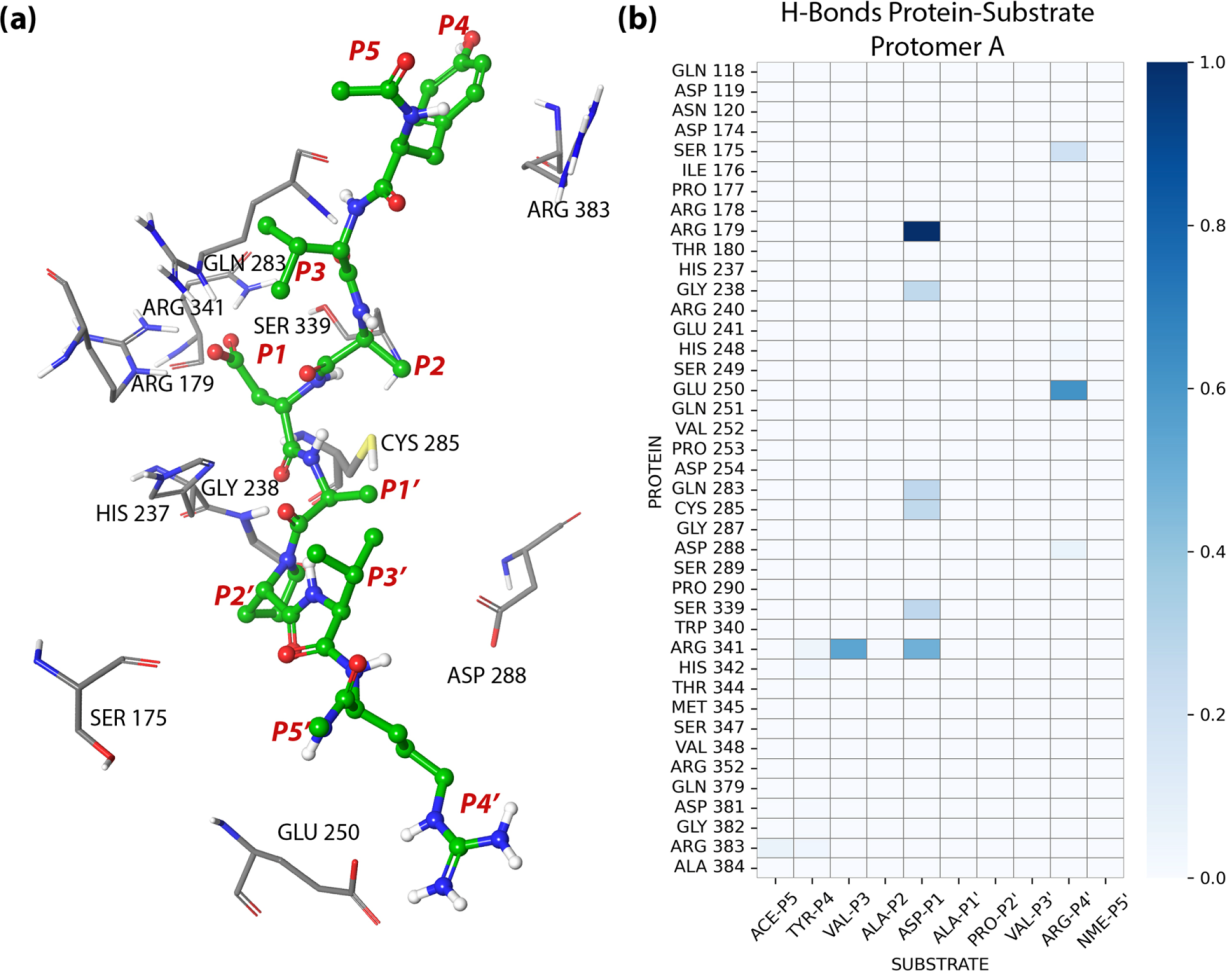
Interactions enzyme–substrate (a) binding pose of the substrate (in balls and rods) in the binding site, the gray residues, in licorice, are those with which it interacts more strongly through hydrogen bonds. (b) Hydrogen-bond interactions between the residues of the substrate and the caspase-1 enzyme found during the three replicas of 1 μs. A hydrogen-bond interaction was counted when the donor–acceptor distance is <3.8 Å and the hydrogen-bond angle is >120°.

Figure 5a shows a representative snapshot of the peptide substrate in the active site of caspase-1 together with a heatmap (Figure 5b) of hydrogen-bond interactions established between the substrate and the enzyme. In this figure, following the Schechter and Berger nomenclature,^68^ the substrate amino acids are named with the letter P numbered from 1 from the scissile bond up to P5 toward the N-terminal region of the substrate, while the amino acids after the scissile bond, toward the C-terminal region, are named P1′, P2′, P3′, and so forth. The specificity of caspase-1 for an aspartate residue at position P1 (Asp-P1) is here reflected by the large number of interactions established between this group and the enzyme. The P1 side chain is placed into the S1 sub-pocket establishing hydrogen bonds with the side chain of residues Arg179, Arg341, and Gln283. The backbone amino group of P1 is hydrogen-bonded to the side chain of Ser339, while its carbonyl carbon group is stabilized by the oxyanion hole, formed by Cys285 proton donor groups (side-chain thiol and backbone amino groups) and the amino group of Gly238. The other residue involved in the scissile peptide bond, Ala-P1′, is surrounded by the side chains of residues Trp340 and Val338, while its mainchain atoms are highly exposed to the solvent. Pro-P2′ occupies the S2′ sub-pocket formed by Ile176, His237, Ile239, and His248, being stabilized mainly by nonpolar interactions. The Val-P3′ residue is exposed to the solvent, and the interactions observed for the Arg-P4′ correspond to its side chain with both Ser175 and Gln250. Moving to the N-terminal side of the substrate, the Ala-P2 fragment is in close contact with Val338 and Trp340 side chains, Val-P3 keeps mainchain–mainchain interactions with Arg341, and the Tyr-P4 residue remains in the S4 sub-pocket formed by His342, Val348, and Arg383. The binding mode described for the substrate agrees with the structures obtained for caspase-1 inhibitors^20,36^ and their simulations.^16^Figure S3 shows the results obtained from the simulation of the Michaelis complex corresponding to the H237A mutant. The set of interactions observed between the substrate and the protein are very similar in both variants, wild type and mutant caspase-1. The mutant establishes a stronger P3-Arg341 interaction than the wild-type enzyme and a weaker P4′-Ser175 one.

**Figure 6:**
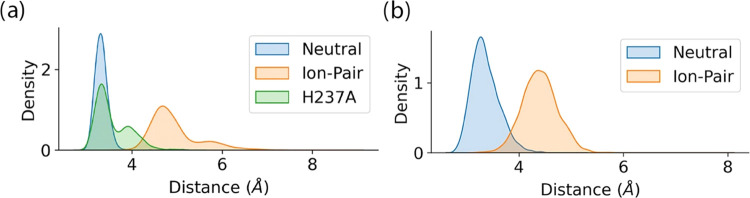
Probability density plots for the distances between (a) the carbonyl carbon atom C(P1) of the substrate and Cys285–Sγ in wild type (neutral and ion-pair catalytic dyad) and H237A caspase-1 and (b) the amide nitrogen atom N(P1′) of the substrate and the His237-Nδ atom in wild type (neutral and ion-pair catalytic dyad) caspase-1.

To test if the choice of a neutral catalytic dyad is adequate to represent the Michaelis complex in caspase-1, we also ran three 1 μs replicas of the system with the catalytic dyad in the IP form, an unprotonated Cys285 and a doubly protonated His237. The RMSD plots shown in Figure S2 correspond to a stable protein–substrate complex in the time scale covered by our simulations, although with larger fluctuations of the substrate in the case of the IP form. Instead, the simulation of inhibitors showed a trend to leave the active site when the catalytic dyad was modeled in the IP state.^16^ The difference is probably due to the stronger interactions established by the enzyme with the natural substrate than with the inhibitors. Although the overall pose of the peptide in the active site with the IP is similar to that described for the neutral catalytic dyad (see Figure S4), some significant differences appear when analyzing the positioning of the scissile peptide bond relative to the catalytic dyad. Figure 6a shows the probability distributions for the distance between the Sγ atom of Cys285 and the electrophilic carbon of the substrate, C(P1), when the dyad is in the IP state and in the neutral state. The complex does not sample configurations compatible with a direct nucleophilic attack of Cys285 to the scissile peptide bond when the catalytic dyad is in the IP form. We also plotted the distance corresponding to the H237A mutant, showing that a large fraction of configurations displays a short distance between the catalytic cysteine and the scissile peptide bond. The distance between the Nδ atom of His237 and the N(P1′) atom of the substrate (Figure 6b) is also substantially larger in the IP form of the wild-type enzyme than in the neutral one, due to the displacement of the charged histidine that now interacts with the side chain of the substrate Asp-P1 group. These simulations point out that the IP state of the catalytic dyad seems not to be compatible with the formation of a reactive complex. A similar conclusion was reached after simulations in other cysteine proteases related to caspases: the legumain^31^ and the RgpB gingipain^32^ cysteine proteases also likely present a neutral catalytic dyad in the Michaelis complex.

### 3.2 Reaction Mechanism

Once the Michaelis complex is analyzed, we studied the reaction mechanism for the proteolysis of the substrate in the active site of caspase-1 using DFT/MM calculations combined with the adaptive string method to explore the multidimensional free energy surface associated with the reaction. Simulations were carried out for both the acylation and deacylation steps in the wild type and H237A mutant versions of caspase-1. We present first the results for the wild type and then for the mutant.

### 3.3 Acylation in Wild-Type Caspase-1

**Figure 7:**
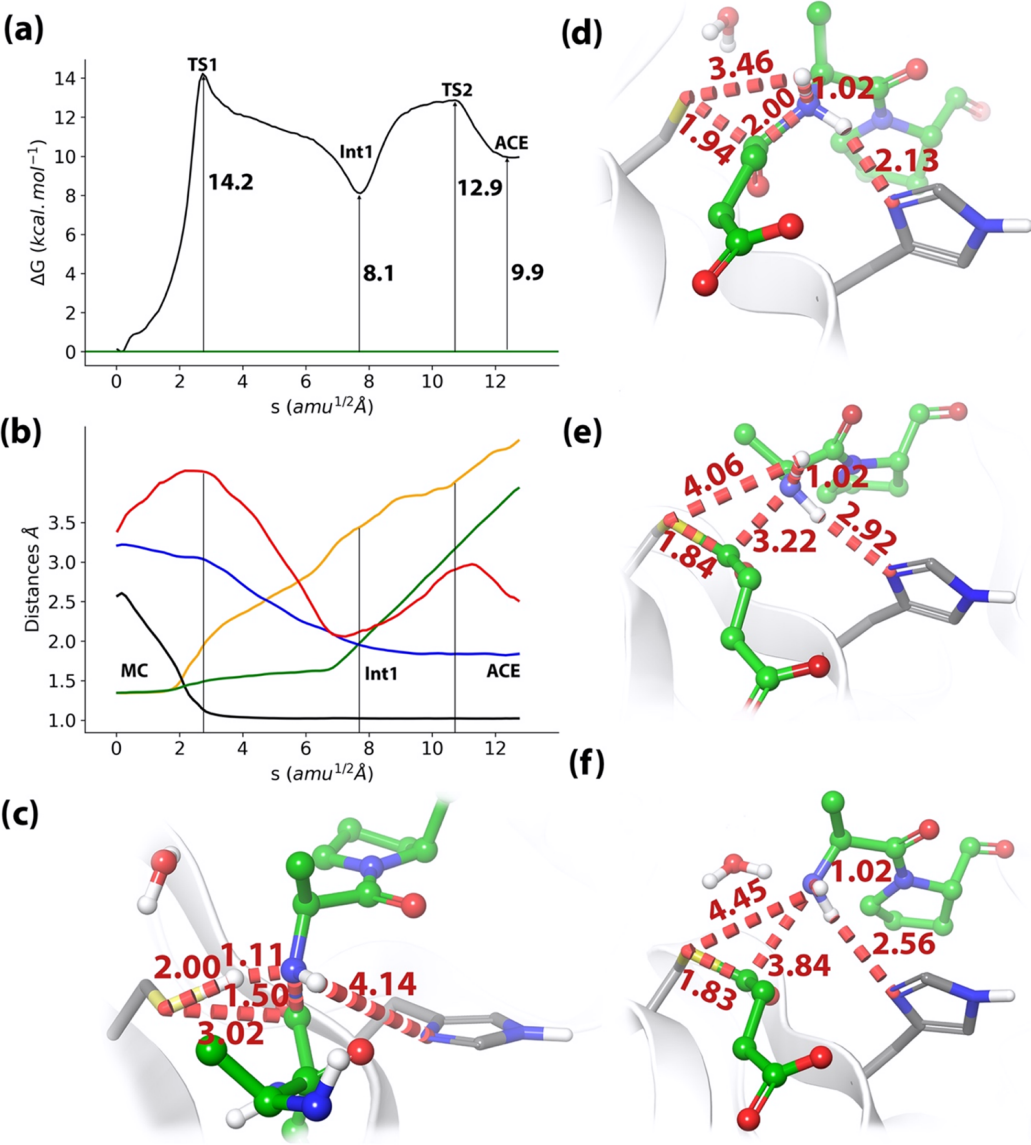
Acylation reaction in wild-type caspase-1. (a) B3LYPD3/MM free energy profile along the path-CV from the Michaelis complex (MC). (b) Evolution of the collective variables (CVs) along the minimum free energy path (MFEP). Sγ–H evolution is presented in yellow line, H–Nε in red, C(P1)–N(P1′) (the scissile peptide bond distance) in green, H–N(P1′) in black, and Sγ–C(P1) in blue. (c) Representation of the TS1 for the acylation process. The values of the distances (in Å) correspond to the CV values on the MFEP. (d) Representation of the intermediate structure. (e) Representation of the TS2, corresponding to the breaking of the C(P1)–N(P1′) scissile peptide bond. (f) Representation of the acyl–enzyme (ACE) complex formed between the enzyme and the P fragment of the peptide, with a water molecule hydrogen-bonded to the N-terminal group of the P′ fragment.

For the acylation step, the multidimensional free energy landscape defined by five CVs (see Figure 4) was explored at the B3LYPD3/MM level. The free energy profile along the MFEP, the evolution of the CVs, and the structure of the stationary states are shown in Figure 7. According to these results, acylation is a stepwise process (see Figure 7a). The first step corresponds to the proton transfer from Cys285–Sγ to the N(P1′) atom, described by CV1 and CV2 (lines in yellow and black in Figure 7b), followed by the nucleophilic attack of Cys285–Sγ on the C(P1) atom (CV4, blue line). The corresponding transition state structure (TS1) is shown in Figure 7c, showing an advanced proton transfer where the distance of the proton to the donor atom (Cys285–Sγ) is larger than to the acceptor (N(P1′)), 2.00 and 1.11 Å, respectively. The free energy barrier associated with this step is 14.2 ± 0.4 kcal·mol^–1^, a value compatible with the experimentally derived free energy barrier for the overall process, 17.9 kcal·mol^–1^. This process is assisted by several interactions contributing to the proton transfer from the catalytic cysteine to the amino leaving group. On the one hand, the acidity of the Cys285–Sγ atom is increased by its hydrogen-bond interaction with the Ser339 side chain (Figure S5a displays the distributions of Cys285–Sγ Ser339–Hγ distances at reactants and at TS1, while Figure S5b shows the structure of TS1 including Ser339). The catalytic role of Ser339 is confirmed by the experimental observation that the S339A mutant shows a 50% reduction in the production of IL-1β with respect to the wild-type enzyme in cell cultures.^36^ On the other hand, the basicity of the N(P1′) atom is increased by an intramolecular hydrogen bond formed with the O(P1′) atom, with a distance of 2.1 Å (see Figure S5c).

The intermediate structure obtained after the proton transfer (Int1) is shown in Figure 7d. This intermediate already shows an increment of the C(P1)–N(P1′) peptide bond distance, as reflected in the evolution of CV3, green line in Figure 7b, that reaches a value of 2.00 Å. Simultaneously, the distance between the C(P1) atoms and the Cys285–Sγ atom is reduced to 1.94 Å. This intermediate is stabilized by the hydrogen-bond interactions formed between the carbonyl oxygen atom of the peptide bond O(P1) and the oxyanion hole, the amide groups of Cys285 and Gly238. The hydrogen-bond distance with the former is drastically reduced from TS1 to Int1, 3.25–2.41 Å respectively, while the distances to the latter remain almost constant, around 2.1 Å. The formation of Int1 is also assisted by the approach of the His237-Nδ atom to N(P1′) up to 2.13 Å (CV5, red line in Figure 7b). This observation suggests that His237 plays a role in the reaction mechanism of the acylation process stabilizing the protonation of the N(P1′) atom. Note that this role can only be played if this residue is found in its neutral form and not in the protonated one.

From Int1 intermediate, the reaction continues until the formation of the acyl–enzyme (ACE, in Figure 7f) through a second TS (TS2, in Figure 7e). During this process, the formation of the Sγ–C(P1) bond is completed, reaching a distance of 1.83 Å (CV4, blue line in Figure 7b), while the peptide bond is completely broken, with the C(P1)–N(P1′) distance increasing up to 3.84 Å (CV3, green line in Figure 7b). Also, the hydrogen bond distance from the amino leaving group to His237 is increased from 2.13 to 2.56 Å (CV5, red line in Figure 7b) as the positive charge on this group is canceled. Our mechanism thus assigns a stabilizing role for His237 during the acylation step, but only for the intermediate state, increasing the distance to the substrate when the thioester acylenzyme complex is formed. The relative free energy barrier of TS2 with respect to the reactants is 12.9 ± 0.6 kcal·mol^–1^, below TS1, while the free energy of the acyl–enzyme intermediate, the starting point for the next reaction step (deacylation), is 9.9 ± 0.8 kcal·mol^–1^.

### 3.4 Deacylation in Wild-Type Caspase-1

The same B3LYPD3/MM level employed to study acylation was also used for the deacylation process, but in this case, the multidimensional free energy landscape for the reaction was defined using seven collective variables, as shown in Figure 4. The deacylation mechanism is carried out by a water molecule that must be activated to break the Sγ–C(P1) acylenzyme bond. A solvent molecule located in the neighborhood of the electrophilic carbon was selected as the hydrolytic water molecule. The radial distribution functions of the oxygen atoms of water molecules around the nitrogen atom of the scissile peptide bond both in the Michaelis and acylenzyme complexes are shown in Figure S6. The selected water molecule was found at a short enough distance from the electrophilic carbon to carry out the nucleophilic attack (around 3.5 Å) and also hydrogen-bonded to the P′-NH_2_ fragment obtained from the acylation. As discussed above, Wilson et al.^36^ proposed that the catalytic histidine deprotonates that water molecule, which then carries out a nucleophilic attack over the C(P1) atom to form a tetrahedral intermediate (see Figure 1). Sulpizi et al.^39^ also suggested that the water molecule becomes activated after a proton transfer to the histidine of the catalytic dyad, which could then transfer the proton to the carbonyl oxygen atom (O(P1)) to form a gem diol intermediate. In our simulations of the acylation product, we found that a water molecule is placed in between the P′-NH_2_ peptide fragment resulting from the acylation step and the acylenzyme, ready to perform the hydrolysis of the Sγ–C(P1) bond. Our proposal offers an alternative reaction mechanism where His237 does not play a key role because the water molecule becomes activated by the terminal amino group of the P′ fragment formed after the acylation step. A similar mechanism for water activation was described in the study of the main protease of the SARS-CoV-2 coronavirus.^57^

**Figure 8:**
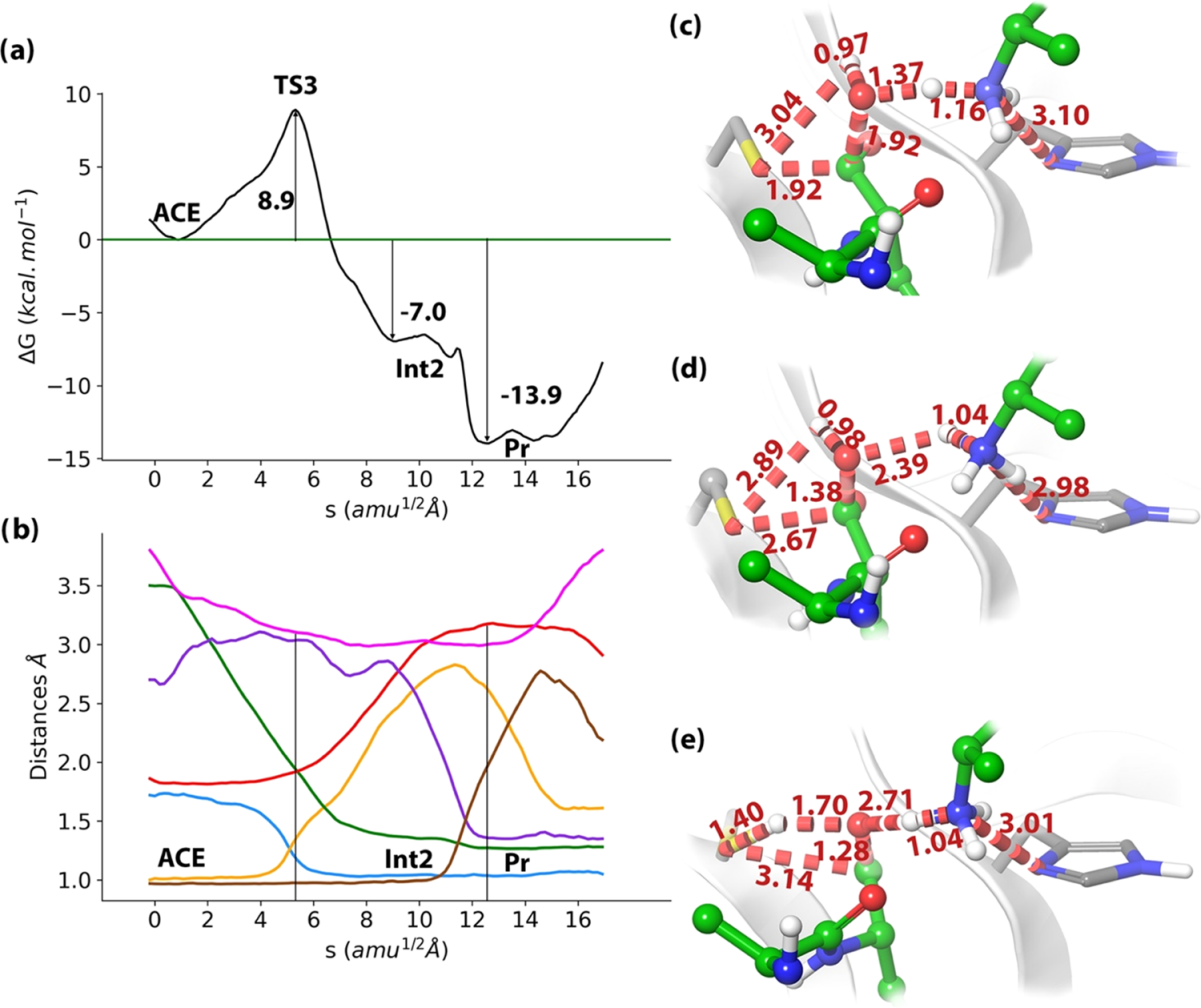
Deacylation reaction in wild-type caspase-1. (a) B3LYPD3/MM free energy profile along the path-CV. (b) Evolution of the selected collective variables along the minimum free energy path. Sγ–Hw2 evolution is shown using a purple line, Nε–N(P1′) in pink, O_w_–C(P1) in green, N(P1′)–Hw1 in blue, Sγ–C(P1) in red, O_w_–Hw1 in yellow, and O_w_–Hw2 in brown. (c) Representation of TS3. The values of the distances (in Å) correspond to the coordinates of the MFEP. (d) Representation intermediate 2 (Int2). (e) Representation of the reaction products (Pr) with the P-COO^–^ and P′-NH_3_^+^ peptide fragments in the active site of the protease.

Analysis of the reaction mechanism for the deacylation process is shown in Figure 8. According to the free energy profile in Figure 8a, the process takes place through a single TS (TS3), which corresponds to the proton transfer from the water molecule to the N(P1′) atom (see the evolution of CV1 and CV2, blue and orange lines in Figure 8b) to yield a P′-NH_3_^+^ peptide fragment. During this process, the oxygen atom of the water molecule (O_w_) approaches the C(P1) atom in the acyl–enzyme, initiating the nucleophilic addition. The O_w_–C(P1) distance is reduced (CV3, green line in Figure 8b) from 3.38 Å in the acylenzyme to 1.92 Å in TS3 (Figure 8c). This activation mechanism for the water molecule offers an alternative to the H237-based activation, in which this residue abstracts the proton from the water molecule. Our proposal could then explain why mutation of this residue (as in H237A) does not completely abolish the enzymatic activity. In addition, one should take into account that terminal amino groups, such as N(P1′), are better bases than the histidine imidazolium ring (the p*K*_a_ values are about 7.7 and 6.6, respectively).^69^ In any case, His237 plays also a secondary role in the deacylation step, establishing a hydrogen-bond interaction with the terminal amino group that enhances its basicity (see the evolution of CV7, pink line in Figure 8b). The free energy of TS3 relative to the acyl–enzyme complex is 8.9 ± 1.1 kcal·mol^–1^. From TS3, the reaction proceeds to the formation of a metastable tetrahedral intermediate (Int2, shown in Figure 8d) where the Sγ–C(P1) bond has been already elongated to 2.67 Å (see CV4, red line in Figure 8b). From this metastable intermediate, the reaction is completed in an almost barrier-free process with the proton transfer from the C(P1)-terminal group to the Sγ atom (see CV5 and CV6, purple and brown lines in Figure 8b) regenerating the catalytic cysteine in its neutral state and yielding the P-peptide fragment with a terminal unprotonated carboxylate (the product is represented in Figure 8e).

Despite the global similarity between the deacylation mechanisms in SARS-CoV-2 3CL protease and in caspase-1, there are important differences related to the different relative orientation between the members of the catalytic dyad in the two enzymes. In the case of the 3CL protease, after the nucleophilic attack of the water molecule to the acylenzyme complex, a high-energy thiodiolate intermediate is formed with a tetrahedral electrophilic carbon atom where the C–Sγ bond is still formed (2.10 Å).^57^ Instead, in caspase-1, the nucleophilic attack directly leads to a more stable intermediate (Int2) where the C–Sγ bond is already broken (2.67 Å). The proximity of the catalytic histidine in 3CL protease makes more difficult the release of the cysteine from the acylenzyme intermediate after the nucleophilic attack by the water molecule, a process that takes place easily in caspase-1. This difference is also translated to the characteristics of TS3 that shows an earlier character for the nucleophilic attack in caspase-1 than in the 3CL protease (with a larger O_w_–C distance, 1.92 vs 1.69 Å, respectively) and that presents a lower activation free energy as measured from the acylenzyme in the case of caspase-1 than in the SARS-CoV-2 3CL protease (8.9 vs 15.6 kcal·mol^–1^).

**Figure 9:**
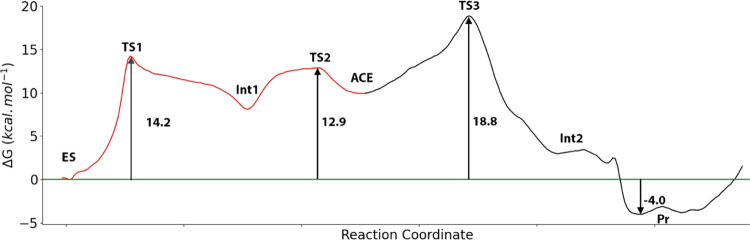
Free energy profile associated with the proteolysis reaction mechanism in the wild-type caspase-1. Acylation step is displayed with red line, while the deacylation step is displayed with black line.

The complete free energy profile for the proteolysis reaction catalyzed by caspase-1 is shown in Figure 9. According to this profile, the deacylation step is the rate-limiting step for the enzymatic reaction with a calculated overall activation free energy of 18.8 ± 1.4 kcal·mol^–1^. This value is in excellent agreement with the value derived from the experimental rate constant (17.9 kcal·mol^–1^). The total reaction process is exergonic presenting a reaction free energy of −4.0 ± 0.9 kcal·mol^–1^. It is worth noticing that in our mechanistic proposal, the two protein fragments resulting from the proteolytic process (P and P′) are obtained in the correct protonation states: P-COO^–^ and P′-NH_3_^+^.

### 3.5 H237A Mutant

In the mechanistic proposal just presented for wild-type caspase-1, the catalytic histidine plays a secondary catalytic role during the reaction, both in the acylation and deacylation processes. In the first one, His237 stabilizes the formation of the intermediate obtained after the proton transfer from the catalytic cysteine to the amino leaving group. In the second one, His237 establishes a hydrogen-bond interaction with the amino group, enhancing its basicity to activate the hydrolyzing water molecule. In any case, the catalytic histidine has a direct participation in a proton transfer event. This mechanistic proposal could then explain the experimental observation that mutations of His237 to Gln, Lys, or even Ala give place to enzyme variants still displaying proteolytic activity.^36^ To check if our proposal is consistent with these experiments, we carried out a study of the reaction mechanism in the H237A variant, using the same computational procedure as before.

**Figure 10:**
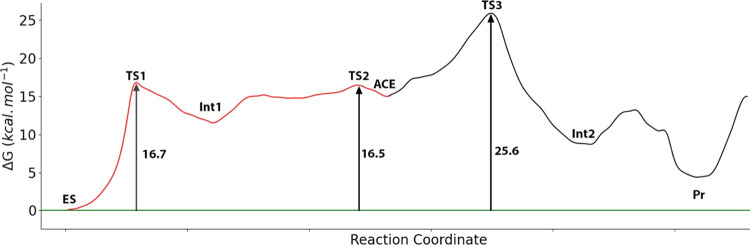
Free energy profile associated with the proteolysis reaction mechanism in the H237A mutant enzyme. Acylation process is displayed in the red line, while the deacylation process is displayed in the black line.

Figure 10 shows the complete free energy profile obtained for the proteolytic process in the H237A variant, while the structures of the stationary states are shown in Figure S7. The geometrical description of the reaction mechanism is similar in the wild type and H237A variants, as deduced from the comparison of the structures corresponding to the stationary states in the wild type and mutant variants of caspase-1 (see Figures 7, 8, and S6). Molecular dynamics simulations at the reactants state show that a water molecule can take the place of the absent catalytic histidine. However, this water molecule is not as effective as the histidine in the stabilization of the intermediate obtained after the proton transfer from the catalytic cysteine to the leaving amino group. This is reflected already in the activation free energy for the proton transfer from Cys285 to the N(P1′) atom that increases from 14.2 kcal·mol^–1^ in the wild-type enzyme to 16.7 kcal·mol^–1^ in the H237A mutant. The relative free energy of the acyl–enzyme intermediate (ACE) also increases from 9.9 to 15.0 kcal·mol^–1^. The rate-limiting step for the overall process in the mutant is also determined by TS3, as in the case of the wild-type enzyme. This TS corresponds to the activation of the hydrolytic water and its nucleophilic attack on the Sγ–C(P1) bond. In the wild-type enzyme, His237 enhances the basicity of the amino terminal group (N(P1′)) abstracting the water proton and then the free energy barrier for this step also increases in the H237A mutant. The total activation free energy barrier increases from 18.8 ± 1.4 kcal·mol^–1^ in the wild-type version to 25.6 ± 2.4 kcal·mol^–1^ in the mutant one. The final proton transfer from the C(P1)-terminal group to the Sγ atom to yield the reaction products presents a small free energy barrier in the mutant, which is not present in the wild-type enzyme. This barrier can be attributed to the positioning of the P′-NH_3_^+^ peptide fragment when H237 is not present, approaching the negatively charged Sγ atom. According to these results, the overall free energy barrier for the proteolysis reaction in the H237A mutant is 6.8 kcal·mol^–1^ larger than for the wild-type version of the enzyme and then the rate constant for the mutant should be about 1.4 × 10^–6^ s^–1^ at 300 K. Experiments have been carried out on cultures with cells transfected with pro-IL-1β encoding cDNA and containing either the wild type or the H237A variant of caspase-1 to produce IL-1β.^36^ Cells containing the mutant enzyme produced about 1% of the IL-1β obtained from cells containing the wild-type variant after 16 h, and 9% after 24 h. While it is difficult to derive the value of the mutant rate constant from these experiments in cells, a rough estimation can be obtained from a simple irreversible first-order kinetic scheme. Assuming an exponential decay of the reactants’ concentration (the observed increase after 16 h rules out the possibility of constant pro-IL-1β concentration) and that the wild-type reaction is completed before 16 h (which seems plausible for a rate constant of 0.78–0.88 s^–1^), the rate constant for the mutant (*k*′) can be derived from the observed IL-1β ratios (*x* = 1 and 9%) after a time period (Δ*t* = 16 and 24 h) to be
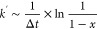
The values obtained are 10^–7^ to 10^–6^ s^–1^, in agreement with the computational estimation. These values must be taken with caution considering the uncertainties associated with the conditions in which the kinetic experiments with cells were carried out and the drastic assumptions made to estimate them.

## 4 Discussion

**Figure 11:**
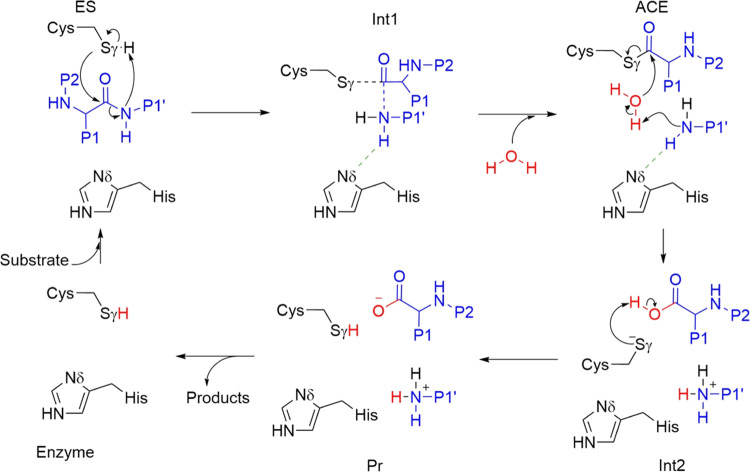
General scheme for the proteolysis reaction in caspase-1.

Exploration of the free energy landscape associated with the proteolysis reaction in caspase-1 shows that the process is composed of two stages: acylation and deacylation, both represented in Figure 11. According to our simulations, acylation is initiated after a proton transfer from the catalytic cysteine to the amide group of the scissile peptide bond. This process is assisted by some key hydrogen-bond interactions: cysteine acidity is increased by a hydrogen bond donated by Ser339, while the basicity of the amide group is increased by an intramolecular hydrogen bond. The intermediate obtained after the proton transfer is stabilized by the oxyanion hole and also by a hydrogen-bond interaction with the catalytic histidine, which is not directly involved as a proton donor or proton acceptor in any step of the process. The intermediate leads to the formation of the acylenzyme complex after breaking the scissile peptide bond. During the deacylation process, a water is activated by the amino terminal group of the peptide fragment liberated during the acylation stage, whose basicity is increased by a hydrogen bond formed with the catalytic histidine. The activated water molecule breaks the carbon–sulfur bond formed between the substrate and the enzyme, regenerating a protonated catalytic cysteine and forming the second peptidic fragment.

Our simulations show that the catalytic dyad in caspases, belonging to the CD clan and C14 family, behaves differently to other cysteine proteases, where the proteolytic activity is developed after the autoionization of the dyad. In the standard mechanism for cysteine proteases, the catalytic cysteine transfers a proton to the histidine, resulting in two charged residues that then carry out the proteolytic process. Formation of the IP increases the nucleophilic character of the catalytic cysteine and enables the catalytic histidine to act as a proton donor to the substrate. Instead, in our mechanistic proposal for caspases, the catalytic cysteine transfers the proton directly to the substrate while the catalytic histidine stabilizes the protonated substrate acting just as a hydrogen-bond acceptor during the acylation and deacylation stages. This mechanism is most probably common for all the CD clan. The same activation mechanism for the catalytic cysteine has been previously proposed for a legumain (CD clan, family C13)^31^ and a gingipain (CD clan, C25 family).^32^ Other enzymes of the same clan share some of the characteristics observed for caspases regarding the catalytic dyad. The structure of the enzyme separase (CD clan, family C50) presents also a large distance between the catalytic dyad (the CysSγ–HisNδ distance is 5.9 Å) preventing also the autoionization mechanism.^70^ Instead, the members of the most common clan in cysteine proteases, the CA clan, present a significantly shorter distance between the catalytic cysteine and histidine (see Figure 2) and would use the standard reaction mechanism involving the formation of an IP at the catalytic dyad. This mechanistic change between clans of cysteine proteases agrees with the different reactivity observed with haloacetate and haloacetamide that suggest that the IP would be characteristic of CA clan and not of CD clan.^71^

Then, the question that arises is why enzymes with the same function and the same catalytic dyad present significant mechanistic differences. We hypothesize that the reason for such a change between clans can be related to the different substrate preferences. To check this hypothesis, we have explored the MEROPS database^72^ (release 12.4),^73^ looking for cysteine proteases with specificity for a charged residue (Asp, Glu, Lys, or Arg) at position P1. The list of enzymes presenting a significant preference (>75%) for a charged residue at P1 is given in Table S2. Most of these enzymes (20 out of 25) belong to the CD clan, while only three belong to the CA clan (one belongs to the PB clan and another remains unassigned). The enzymes of the CD clan listed in Table S2 show a large preference for aspartate, lysine, or arginine at P1 position. The binding of this charged residue in the active site requires the presence of oppositely charged residues in the S1 site (i.e., Arg179 and Arg341 in caspase-1). The presence of these charges could impose an extra free energy penalty for the creation of an IP in the catalytic dyad, due to electrostatic repulsions. The mechanistic difference observed between CA and CD clans could then have been triggered by the need to avoid this extra free energy cost in the formation of an IP.

## 5 Conclusions

We have here addressed the computational analysis of the reaction mechanism in human caspase-1, an enzyme in charge of the transformation of the pro-inflammatory cytokine pro-interleukin-1β (pro-IL-1β) into its IL-1β active form, a key process in the inflammatory response and in many diseases, such as Alzheimer’s disease. Caspase-1 is a cysteine protease, a group of enzymes that catalyze proteolysis using a catalytic dyad constituted by cysteine and histidine. The structure of the active site of caspases, including that of caspase-1, seems incompatible with the standard mechanistic proposal for cysteine proteases, where the catalytic histidine abstract the proton from the cysteine, before or after formation of the Michaelis complex. In caspases, the distance between the two catalytic residues is larger and the substrate is placed between the two catalytic residues, preventing a direct proton transfer between them. Experimental mutagenesis studies in caspase-1 also show that the enzymatic activity is not completely abolished after mutation of the catalytic histidine into alanine.

For the first time, we have been able to solve the mechanistic puzzle of caspases using a combination of classical and hybrid DFT/MM simulations that provide results in excellent agreement with experiments. In our proposal, the catalytic cysteine transfers the proton to the substrate before the nucleophilic attack. The calculated activation free energy is in very good agreement with the value derived from the experimental rate constant, 18.8 vs 17.9 kcal·mol^–1^, which supports our mechanistic proposal. Experimental mutagenic studies carried out for caspase-1 can be interpreted in the light of our mechanistic proposal. The S339A mutant shows a 50% reduction in the activity with respect to the wild-type enzyme, as determined by the ability to produce IL-1β in cell cultures, while the H237A mutant shows a smaller but non-negligible activity.^36^ As shown above, these two residues facilitate the acylation step, assisting the proton transfer from the catalytic cysteine to the leaving group. His237 also assists the deacylation step enhancing the basicity of the amino group activating the proteolytic water molecule. Importantly, the catalytic histidine does not directly participate in any proton transfer event during proteolysis. We performed DFT/MM simulations for the H237A mutant that shows a significant increase in the activation free energy with respect to the wild type, by about 6.8 kcal·mol^–1^. The calculated barrier, 25.6 kcal·mol^–1^, is compatible with the reduced, but not abolished, proteolytic activity observed experimentally.

The reaction mechanism presented here for caspase-1 and summarized in Figure 11 is probably valid for the rest of the members of the CD clan of cysteine proteases. The origin of the mechanistic difference with respect to other clans of cysteine proteases could be due to the preference of the CD clan for charged residues at the P1 position, just before the targeted peptidic bond. All in all, our study shows how multiscale simulations can be used to elucidate the reaction mechanism even in complex cases, such as the caspase family, provided that an adequate combination of methods is selected. Our proposal not only agrees with experimental data but also provides a rationalization for the mechanistic change described within the family of cysteine proteases. Finally, we have identified several key structures along the reaction path that can be used as templates for the design of potential inhibitors of the activity of caspase-1. The mechanistic peculiarities found for this family of enzymes can be exploited to design more specific inhibitors of caspase activity.

## Supporting Information Available

Analysis of distances on crystallographic structures of caspase-1; representation of the QM region and CVs in the H237A mutant; RMSD in the MD simulations; analysis of hydrogen-bond interactions in H237A mutant; binding pose of the substrate in caspase-1 with ionized catalytic dyad; distribution of hydrogen-bond distances between Ser339 and Cys285; radial distribution functions of water molecules around the nitrogen atom of the scissile peptide bond; representation of stationary structures along the MFEP in the H237A mutant; and list of cysteine proteases with preference for charged residues at P1 (PDF)
