# Supplementary material for: Unveiling the Mechanistic Singularities of Caspases: A Computational Analysis of the Reaction Mechanism in Human Caspase-1
Source: ACS Catal. 2023 Mar 15;13(7):4348–61. doi: 10.1021/acscatal.3c00037 (PMC10088814; doi:10.1021/acscatal.3c00037)
Supplement: Supplementary file 1 — cs3c00037_si_001.pdf [file cs3c00037_si_001.pdf]

Supplementary information

Unveiling the Mechanistic Singularities of Caspases. A Computational Analysis of the Reaction Mechanism in Human Caspase-1

Carlos A. Ramos-Guzmán[a,b], J. Javier Ruiz-Pernía[a], Kirill Zinovjev[a]*, Iñaki Tuñón[a]*

[a]Departamento de Química Física, Universidad de Valencia, 46100 Burjassot (Spain)
[b]Instituto de Materiales Avanzados, Universidad Jaume I, 12071 Castelló (Spain)

*To whom correspondence should be addressed:
ignacio.tunon@uv.es
kirill.zinovjev@uv.es

Table S1. Distances (in Å) between the P1-carbonyl oxygen atom with His237Nδ, Cys285N and Gly238 N atoms and between His237Nδ and Cys285Sγ, measured from the crystallographic structures of covalently inhibited caspase-1 enzymes.

PDB	His237Nδ-O	Cys285N-O	Gly238N-O	His237Nδ–SγCys285
1IBC	2.51	3.97	3.88	5.06
1ICE	2.65	3.85	4.02	5.32
3NS7	2.68	4.08	4.09	5.30
1RWP	2.78	3.52	3.64	5.24
1RWN	2.81	3.58	3.51	5.26
1BMQ	2.83	3.57	3.58	5.23
5MTK	2.83	4.08	4.87	4.88
1RWX	2.84	3.45	3.50	5.19
1RWW	3.00	3.43	3.31	5.28
6PZP	3.11	3.39	3.37	5.54
1RWM	3.21	3.41	3.28	5.30
5MMV	3.26	3.16	4.16	5.32
6F6R	4.27	2.76	3.91	6.08

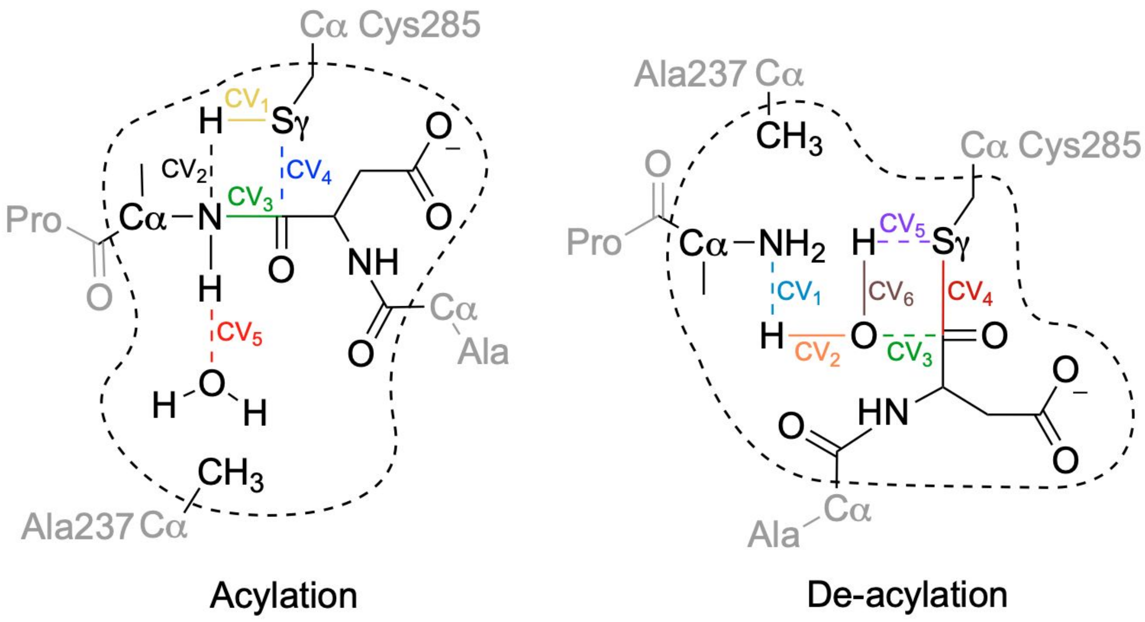

Figure S1. QM/MM partitioning scheme used to evaluate the acylation and deacylation rection mechanism proposals for the H237A mutant caspase-1. Atoms inside the dashed regions were treated at the QM level while everything else was evaluated at the MM level. The set of CVs describing the relevant changes during the chemical steps are also displayed.

a)

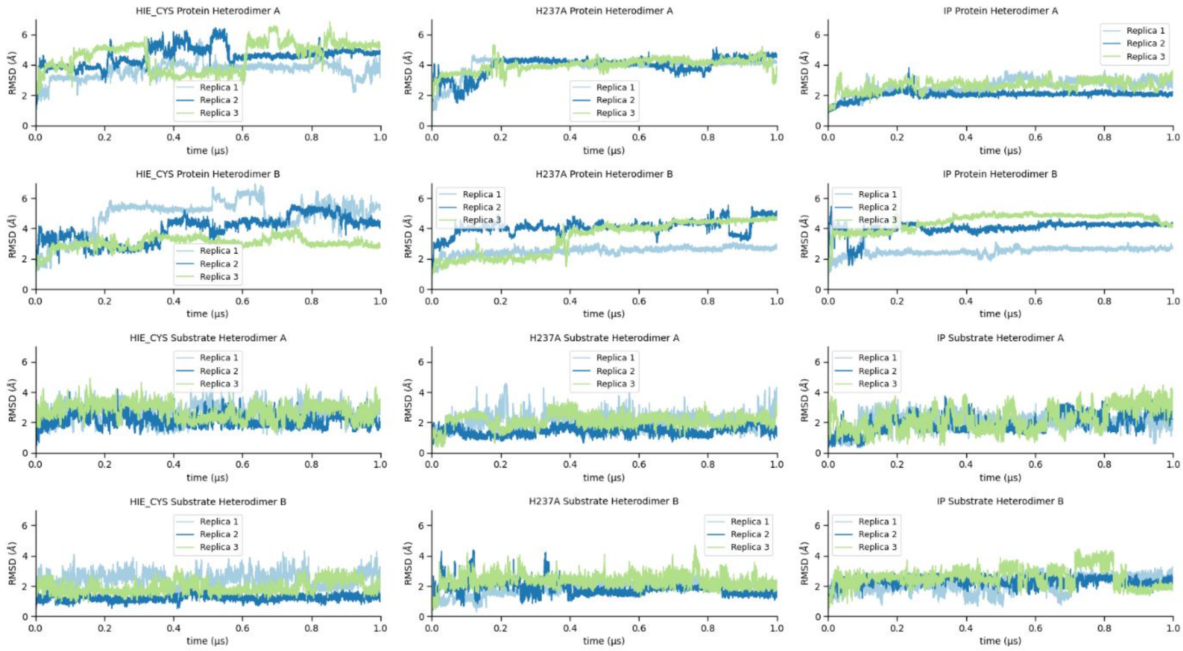

b)

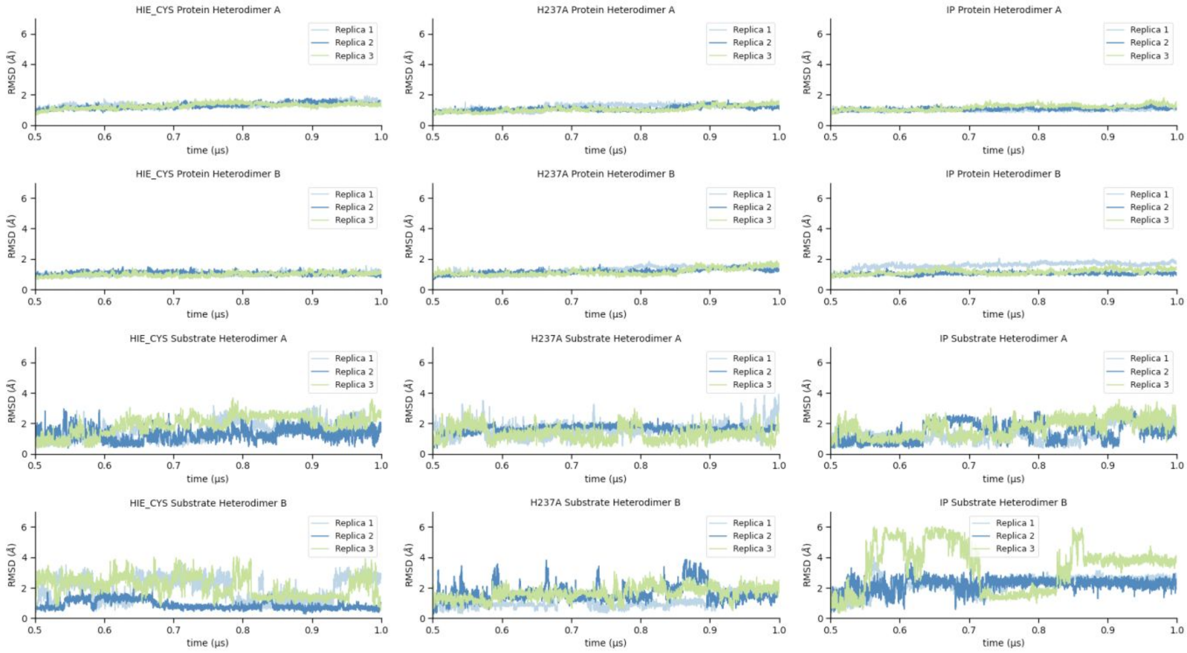

Figure S2. (a) Plot of the RMSD values for caspase-1 wild type protein and the H237 mutant with respect to the intial structures. Each plot represents the 3 replicas of 1 μs of the Michaelis Complex with a peptide mimetic substrate. The RMSD has been computed using all non-hydrogen atoms taking as reference the initial structure prepared for the simulations. For each replica, the RMSD of each monomer and each of the two substrates is shown. **(b)** Plot of the RMSD values calculated with respect to the frame obtained at 500 ns of the trajectories. In this case the RMSD was calculated considering only the structured parts of the enzyme, this is including residues 136-297 of β subunits and residues 317-404 of the α subunits.

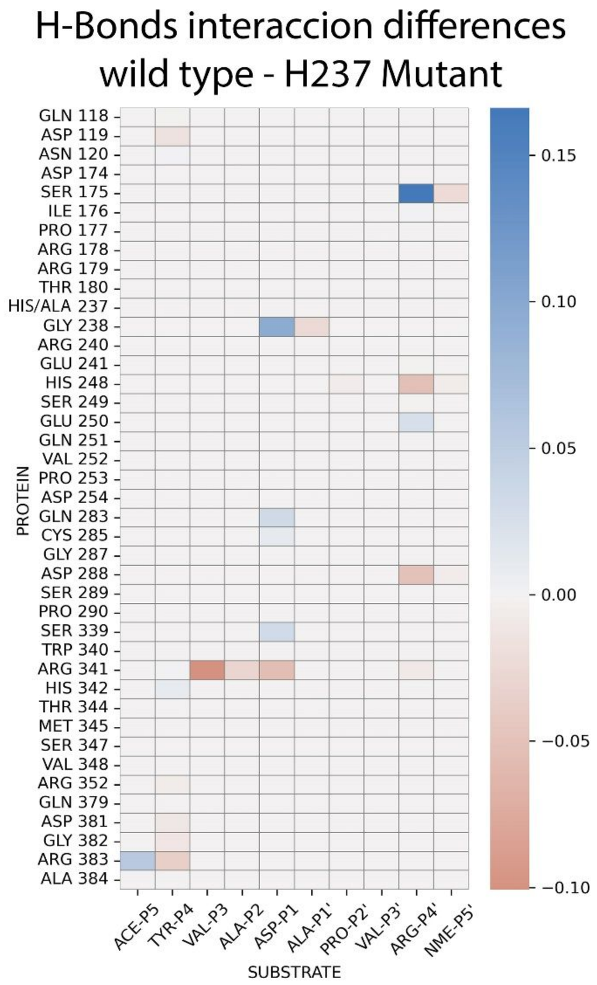

Figure S3. Differences in the frequency of hydrogen bond interactions between the substrate and the protein in the wild type and H237A mutant caspase-1.

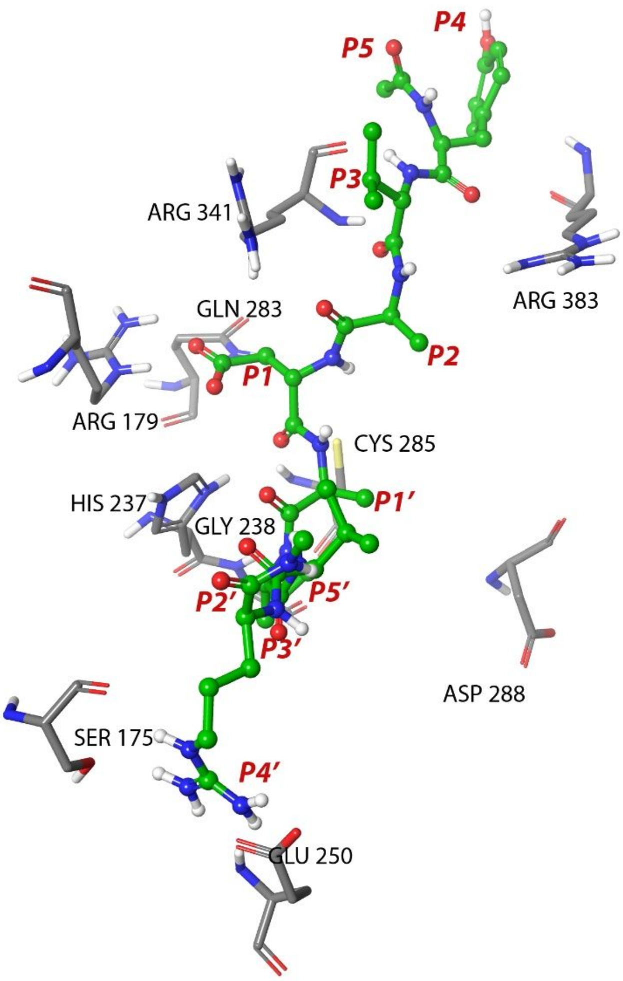

Figure S4. Pose of the substrate in the active site of the caspase-1 enzyme when the catalytic dyad is in its ion-pair configuration.

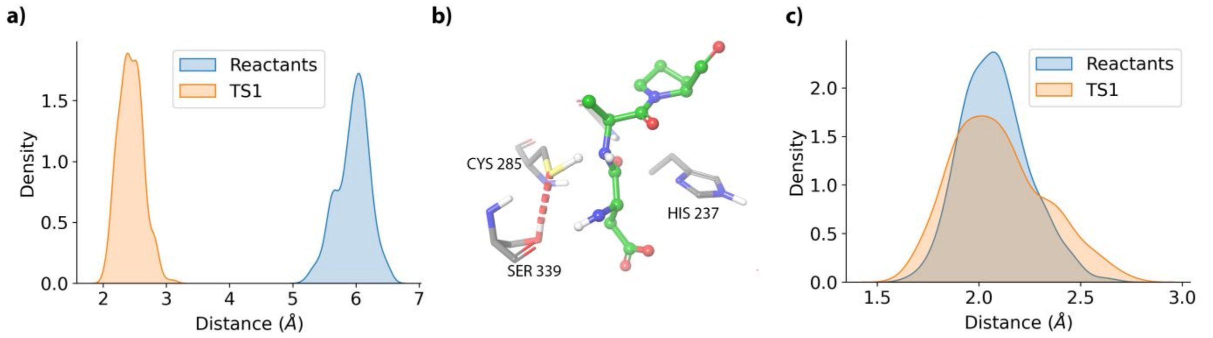

Figure S5. (a) Probability distribution for the distance between atoms Cys285-Sγ and Ser339-Hγ in the reactants (blue) and transition state (TS1, in orange) of the acylation step in caspase-1 and (b) Representation of TS1 highlighting the hydrogen bond between Ser339 and Cys285; (c) Probability distribution for the distance between atoms HN(P1′) and O(P1′) in the reactants (blue) and transition state (TS1, in orange) of the acylation step in caspase-1.

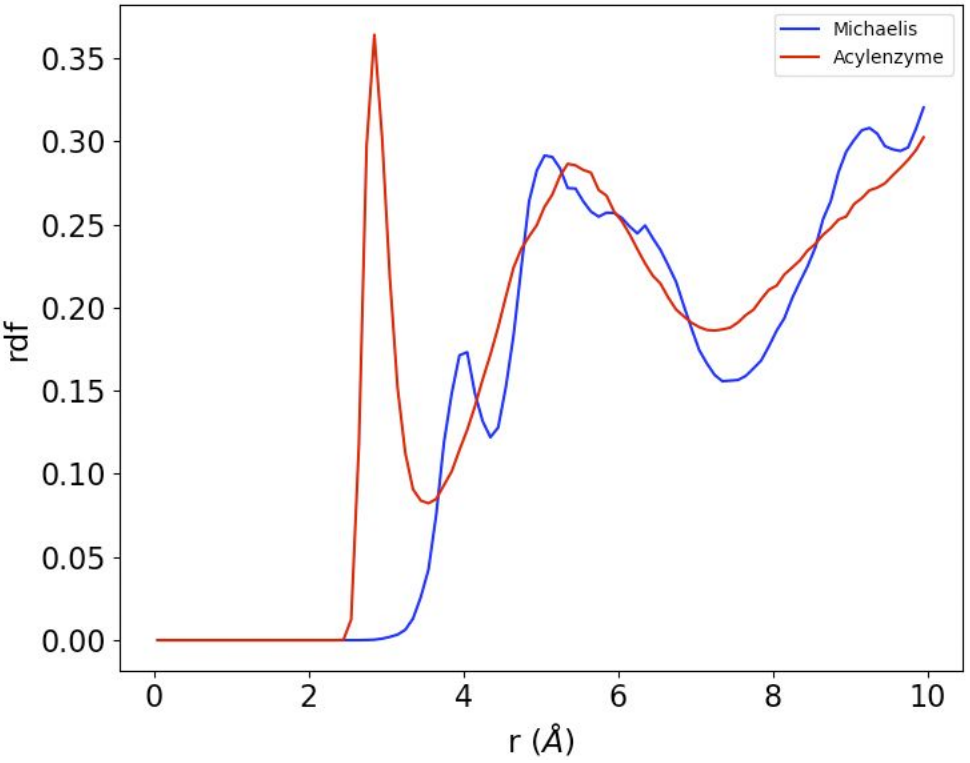

Figure S6. Radial distribution functions of water oxygen atoms around the nitrogen atom of the scissile peptide bond in the Michaelis and in the acylenzyme complexes.

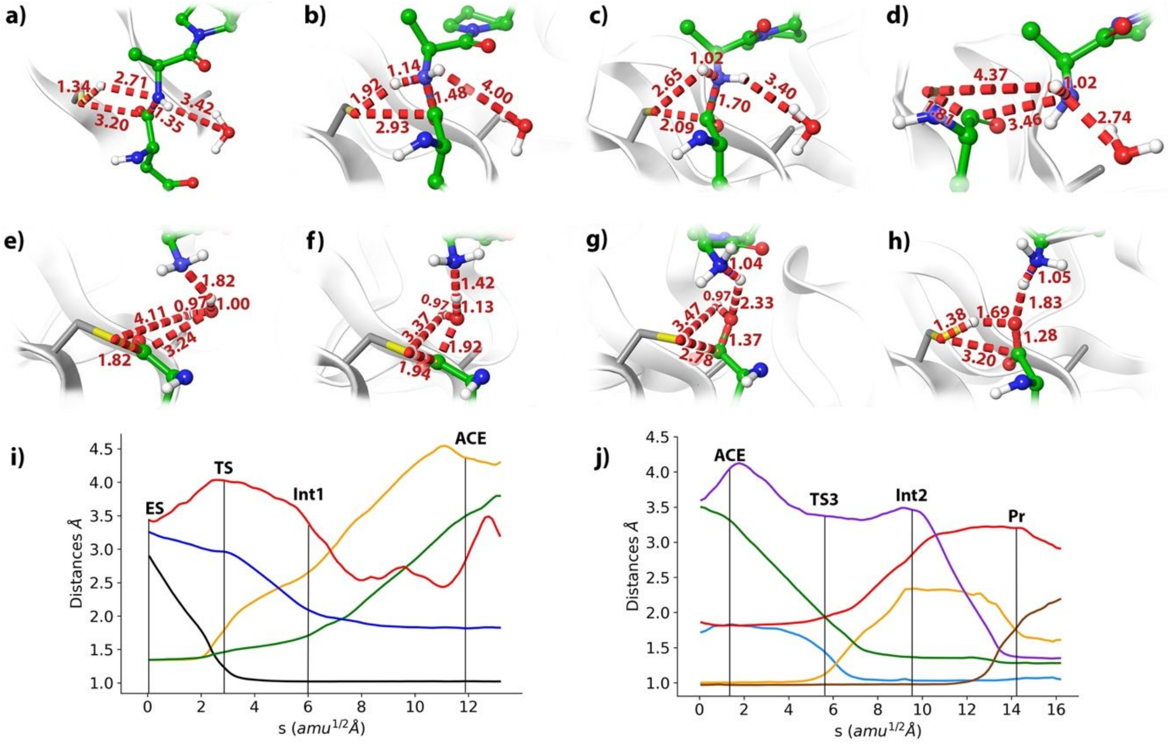

Figure S7. Stationary states obtained for the proteolysis reaction in the H237A variant of caspase-1. The values of the distances (in Å) correspond to the CV values on the MFEP. (a) Michaelis Complex. (b) TS1. (c) Int1. (d) TS2. (e) Acyl-Enzyme (ACE). (f) TS3. (g) Int2. (h) Products. (i) Evolution of the collective variables (CVs) along the minimum free energy path (MFEP) of the acylation step in H237A mutant. Color code correspond to Figure S1. (j) Evolution of the collective variables (CVs) along the minimum free energy path (MFEP) of the deacylation step in H237A mutant. Color code correspond to Figure S1.

Table S2. List of cysteine proteases with large preference (> 75%) for a charged residue at position P1, according to the MEROPS database.

MEROPS ID.	name	P1 preference	% preference	Clan, Family
C14.001	caspase-1	ASP	84	CD, C14
C14.002	CED-3 peptidase	ASP	100	CD, C14
C14.003	caspase-3	ASP	98	CD, C14
C14.004	caspase-7	ASP	89	CD, C14
C14.005	caspase-6	ASP	100	CD, C14
C14.006	caspase-2	ASP	100	CD, C14
C14.007	caspase-4	ASP	100	CD, C14
C14.009	caspase-8	ASP	100	CD, C14
C14.010	caspase-9	ASP	85	CD, C14
C14.011	caspase-10	ASP	100	CD, C14
C14.018	caspase-14	ASP	100	CD, C14
C14.019	caspase DRONC	ASP	81	CD, C14
C25.002	gingipain K	LYS	97	CD, C25
C01.050	histolysain	ARG	81	CA, C1
C01.074	CPB peptidase	ARG	82	CA, C1
C01.115	fascipain B	ARG	80	CA, C1
C14.026	paracaspase	ARG	100	CD, C14
C14.035	metacaspase Yca1	ARG	81	CD, C14
C14.043	metacaspase Ld	ARG	96	CD, C14
C14.044	metacaspase TbMCA2	ARG	81	CD, C14
C25.001	gingipain RgpA	ARG	92	CD, C25
C25.003	gingipain RgpB	ARG	75	CD, C25
C50.001	separase (yeast-type)	ARG	100	CD, C50
C69.002	arginine aminopeptidase	ARG	100	PB, C69
C9G.030	bothropain	ARG	75	Unassigned
